# Silencing of genes involved in *Anaplasma marginale*-tick interactions affects the pathogen developmental cycle in *Dermacentor variabilis*

**DOI:** 10.1186/1471-213X-9-42

**Published:** 2009-07-16

**Authors:** Katherine M Kocan, Zorica Zivkovic, Edmour F Blouin, Victoria Naranjo, Consuelo Almazán, Ruchira Mitra, José de la Fuente

**Affiliations:** 1 Department of Veterinary Pathobiology, Center for Veterinary Health Sciences, Oklahoma State University, Stillwater, OK 74078, USA; 2 Utrecht Centre for Tick-borne Diseases (UCTD), Department of Infectious Diseases and Immunology, Faculty of Veterinary Medicine, Utrecht University, Yalelaan 1, 3584CL, Utrecht, The Netherlands; 3 Instituto de Investigación en Recursos Cinegéticos IREC (CSIC-UCLM-JCCM), Ronda de Toledo s/n, 13071 Ciudad Real, Spain; 4 Facultad de Medicina Veterinaria y Zootecnia, Universidad Autónoma de Tamaulipas, Km. 5 carretera Victoria-Mante, CP 87000 Cd. Victoria, Tamaulipas, Mexico

## Abstract

**Background:**

The cattle pathogen, *Anaplasma marginale*, undergoes a developmental cycle in ticks that begins in gut cells. Transmission to cattle occurs from salivary glands during a second tick feeding. At each site of development two forms of *A. marginale *(reticulated and dense) occur within a parasitophorous vacuole in the host cell cytoplasm. However, the role of tick genes in pathogen development is unknown. Four genes, found in previous studies to be differentially expressed in *Dermacentor variabilis *ticks in response to infection with *A. marginale*, were silenced by RNA interference (RNAi) to determine the effect of silencing on the *A. marginale *developmental cycle. These four genes encoded for putative glutathione S-transferase (GST), salivary selenoprotein M (SelM), H+ transporting lysosomal vacuolar proton pump (vATPase) and subolesin.

**Results:**

The impact of gene knockdown on *A. marginale *tick infections, both after acquiring infection and after a second transmission feeding, was determined and studied by light microscopy. Silencing of these genes had a different impact on *A. marginale *development in different tick tissues by affecting infection levels, the densities of colonies containing reticulated or dense forms and tissue morphology. Salivary gland infections were not seen in any of the gene-silenced ticks, raising the question of whether these ticks were able to transmit the pathogen.

**Conclusion:**

The results of this RNAi and light microscopic analyses of tick tissues infected with *A. marginale *after the silencing of genes functionally important for pathogen development suggest a role for these molecules during pathogen life cycle in ticks.

## Background

Ticks transmit pathogens that impact both human and animal health [1]. Of these tick-borne pathogens, those belonging to the genus *Anaplasma *(Rickettsiales: Anaplasmataceae) are obligate intracellular organisms found exclusively within parasitophorous vacuoles in the cytoplasm of both vertebrate and tick host cells [2]. The type species, *A. marginale*, causes the economically important cattle disease bovine anaplasmosis [2]. In the United States, *A. marginale *is vectored by *Dermacentor variabilis, D. andersoni*, and *D. albipictus *[2,3].

The life cycle of *A. marginale *in the tick vector is complex and coordinated with tick feeding cycle [4-6]. Bovine erythrocytes infected with *A. marginale *are ingested by ticks in the bloodmeal and the first site of infection in ticks is gut and Malpighian tubule cells. *A. marginale *then infects and develops in salivary glands, the site of transmission to the vertebrate host. Gut muscle and fat body cells may also become infected with *A. marginale *during tick feeding.

Two stages of *A. marginale *occur within a parasitophorous vacuole in the tick cell cytoplasm. The first form of *A. marginale *seen within colonies is the reticulated (vegetative) form (RF) that divides by binary fission and results in formation of large colonies that may contain hundreds of organisms. The RFs then transform into the dense form (DF) which can survive for a short time outside of cells and is the infective form. This developmental cycle occurs at every site of *A. marginale *development in ticks.

The evolution of ticks and the pathogens that they transmit have co-evolved molecular interactions that mediate their development and survival [7], and these interactions involve genetic traits of both the tick and the pathogen. Recently, a functional genomics approach was used to discover genes/proteins that are differentially expressed in tick cells in response to infection with *A. marginale *[7]. In these studies, 4 genes found to be downregulated after RNA interference (RNAi) affected *A. marginale *infection levels in *D. variabilis *guts and/or salivary glands. These four genes encoded for putative glutathione S-transferase (GST), salivary selenoprotein M (SelM), H+ transporting lysosomal vacuolar proton pump (vATPase) and subolesin. The results of these experiments further confirmed that a molecular mechanism occurs by which tick cell gene expression mediates the *A. marginale *developmental cycle and trafficking through ticks [7].

In this study, we characterized the effect of silencing GST, SelM, vATPase and subolesin genes by RNAi on *A. marginale *development and infection levels in *D. variabilis *by quantitative PCR and light microscopy. The analysis was conducted in ticks after acquisition feeding (AF) and transmission feeding (TF) to characterize the effect on gene expression during pathogen trafficking from guts during AF to salivary glands and other tissues after TF [4,5]. The results demonstrated that gene knockdown reduced the number of RF- and DF-containing colonies in various tick tissues with implications for pathogen replication, development and transmission in ticks, and suggested that these genes may be good targets for development of a new generation of pathogen transmission-blocking vaccines for control of bovine anaplasmosis directed toward reducing exposure of vertebrate hosts to *A. marginale*.

## Results

### Confirmation of RNAi of tick genes and *A. marginale *infection levels in ticks

The effect of RNAi on GST, SelM and subolesin gene silencing was confirmed in ticks after AF and TF (Table 1). Silencing the expression of genes encoding for putative GST, vATPase, SelM and subolesin resulted in statistically significant differences in the *A. marginale *infection levels in guts and/or salivary glands when compared to saline-injected controls (Table 2). In ticks in which the expression of putative GST was silenced, *A. marginale *infection was inhibited both in tick guts after AF and in salivary glands after TF. When putative vATPase dsRNA was injected, *A. marginale *infection was inhibited in tick guts after AF but the pathogens were still able to infect and multiply in the salivary glands after TF. The RNAi of salivary SelM expression resulted in the inhibition of pathogen infection and/or multiplication in tick salivary glands after TF. As reported previously [8], subolesin RNAi affected *A. marginale *infection of tick salivary glands after TF. In all cases, infection levels were not affected in guts after TF (Table 2).

**Table 1:** Expression silencing of selected genes in *D. variabilis *male guts and salivary glands after RNAi.

**Experimental group**	**Silencing in guts after AF (% ± SD)**	**Silencing in guts after TF (% ± SD)**	**Silencing in salivary glands after TF (% ± SD)**
GST	81.2.6 ± 12.4*	100 ± 0.0*	100 ± 0.4*
vATPase	ND	ND	ND
SelM	74.1 ± 17.3*	100 ± 0.0*	74.0 ± 25.2*
Subolesin	90.0 ± 21.4*	99.7 ± 0.6*	99.4 ± 0.0*

The silencing of gene expression was analyzed in *D. variabilis *male ticks after RNAi. Salivary glands and/or guts were dissected from 5 ticks after AF and TF and mRNA levels were analyzed by real time RT-PCR. Percent reduction in transcript levels relative to that in tissues from control ticks were averaged over five replicate samples and expressed as average ± SD. Significance was determined by comparing mRNA levels between dsRNA and saline injected control ticks by Student's t-test (*P ≤ 0.05). Abbreviation: ND, not done because RT-PCR conditions could not be established.

**Table 2:** *A. marginale *infection levels in *D. variabilis *male guts and salivary glands after RNAi of selected tick genes.

**Experimental group**	**Infection levels in guts after AF (*A. marginale*/tick ± SE)**	**Infection levels in guts after TF (*A. marginale */tick ± SE)**	**Infection levels in salivary glands after TF (*A. marginale/*tick ± SE)**
GST	5 ± 15*	99,060 ± 68462	2 ± 0*
vATPase	81 ± 5*	795 ± 227	247 ± 205
SelM	389,095 ± 282048	1,451 ± 443	2 ± 0*
Subolesin	814 ± 122	1,517 ± 1025	2 ± 0*
Saline control	40,579 ± 6993	28,252 ± 27788	287 ± 144

The *A. marginale *infection levels were analyzed in *D. variabilis *male ticks after RNAi. Salivary glands and/or guts were dissected from 5 ticks after AF and TF and DNA was used for quantitative *msp4 *PCR to determine *A. marginale *infection levels. Infection levels in tick guts and salivary glands were expressed as average ± SE and compared between dsRNA and saline injected ticks by Student's t-test (*P ≤ 0.05).

### Light microscopy analysis of *A. marginale *colonies in tick tissues

**Figure 1:**
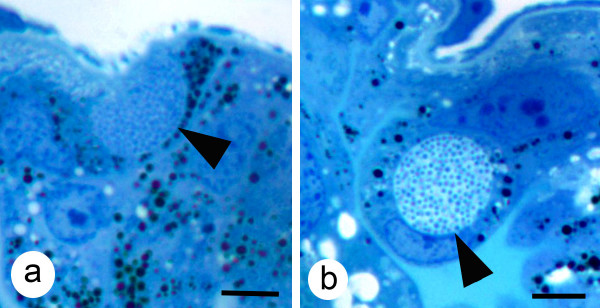
**Light photomicrographs of colonies of *A. marginale *in the gut of male *D. variabilis***. (a) A colony containing reticulated forms (arrow) of *A. marginale *and (b) a gut cell containing a colony with dense forms (arrow). Mallory's stain, Bar = 5 μm.

**Figure 2:**
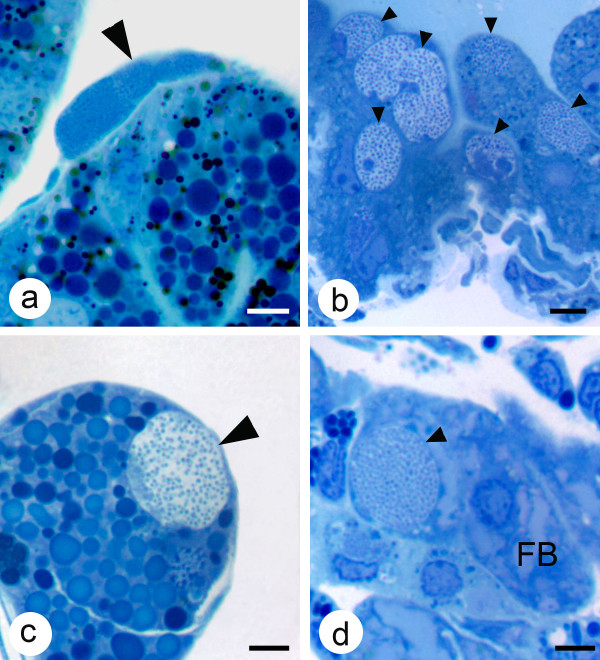
**Light photomicrographs of colonies of *A. marginale *in various tissues of male *D. variabilis***. (a) A colony of *A. marginale *in a gut muscle cell (large arrow); (b) several colonies (small arrows) in Malpighian tubules cells; (c) a colony in a salivary gland cell (large arrow) and (d) a colony of *A. marginale *(small arrow) in a fat body cell. Mallory's stain, bar = 5 μm.

Colonies of *A. marginale *containing RFs or DFs were easily recognizable with light microscopy (Fig. 1) The main site of *A. marginale *infection in ticks after AF was in the gut and Malpighian tubule cells (Figs. 1 and 2), while after TF colonies were also seen in gut muscle, salivary gland acinar and fat body cells (Fig. 2). Quantitative analysis of *A. marginale *colonies in dsRNA-injected ticks demonstrated that gene knockdown by RNAi significantly reduced the density of RFs in the gut of subolesin dsRNA-injected ticks after both AF and TF when compared to controls (Table 3). The density of DFs after AF was significantly lower in guts of ticks injected with GST dsRNA and in guts of ticks injected with subolesin dsRNA after TF (Table 3). In contrast, the density of RFs was significantly higher in guts of ticks injected with SelM dsRNA after AF and TF and the density of DFs was significantly higher in vATPase dsRNA-injected ticks after TF (Table 3). In all silenced ticks, *A. marginale *colonies were not seen in salivary glands after AF or TF; infection of salivary glands was seen only in the control ticks after TF (Table 3).

**Table 3:** Quantitative analysis of *A. marginale *colony densities in *D. variabilis *guts and salivary glands after gene knockdown by RNAi.

	**Tick genes silenced by RNAi**				
	
**Tissue/colonies containing RF or DF**	**GST**	**SelM**	**vATPase**	**Subolesin**	**Control**
**Ticks collected after AF**					

Gut/RF	0.27 ± 0.24	0.85 ± 0.31*	0.62 ± 0.57	0.00 ± 0.00*	0.28 ± 0.20

Gut/DF	0.07 ± 0.01*	0.26 ± 0.26	0.15 ± 0.12	0.17 ± 0.06	0.18 ± 0.13

Salivary glands/RF	0.00 ± 0.00	0.00 ± 0.00	0.00 ± 0.00	0.00 ± 0.00	0.00 ± 0.00

Salivary glands/DF	0.00 ± 0.00	0.00 ± 0.00	0.00 ± 0.00	0.00 ± 0.00	0.00 ± 0.00

**Ticks collected after TF**					

Gut/RF	1.00 ± 0.72	1.43 ± 1.29*	0.63 ± 0.47	0.04 ± 0.01*	0.75 ± 0.59

Gut/DF	0.29 ± 0.23	0.53 ± 0.48	2.62 ± 2.31*	0.04 ± 0.03*	0.32 ± 0.25

Salivary glands/RF	0.00 ± 0.00*	0.00 ± 0.00*	0.00 ± 0.00*	0.00 ± 0.00*	0.003 ± 0.001

Salivary glands/DF	0.00 ± 0.00*	0.00 ± 0.00*	0.00 ± 0.00*	0.00 ± 0.00*	0.01 ± 0.01

The density of *A. marginale *reticulated forms (RF) and dense forms (DF) containing colonies (average ± SD) was calculated for tick gut and salivary gland (sg) sections after acquisition feeding (AF) and transmission feeding (TF) and compared between dsRNA-injected and control ticks by Student's t-test with unequal variance (*P < 0.05). The values were underlined when gene knockdown resulted in higher RF or DF when compared to controls.

Overall, the qualitative analysis of *A. marginale *in *D. variabilis *silenced ticks resulted in the reduction of colonies in gut muscle, Malpighian tubule and fat body cells after TF as compared to the controls (Table 4). Only subolesin dsRNA-injected ticks showed a reduction of *A. marginale *colonies in the malpighian tubules after AF (Table 4). Interestingly, an increase in the number of fat body colonies was seen in GST dsRNA-injected ticks after TF, suggesting that the silencing of this gene enhanced infection of fat body cells and thus represented a shift in the *A. marginale *tick developmental cycle (Table 4).

**Table 4:** Qualitative analysis of *A. marginale *colonies in gut muscle, Malpighian tubule and fat body and tissue degeneration in *D. variabilis *after gene knockdown by RNAi.

	**Genes silenced by RNAi**				
	
**Collection time/tissue**	**GST**	**SelM**	**vATPase**	**Subolesin**	**Control**
AF/GM	-	-	-	-	-

AF/MT	++	++	++	(-)	++

AF/FB	-	-	-	-	-

TF/GM	(++)	+++	(++)	(-)	+++

TF/MT	(++)	(++)	(++)	(-)	+++

TF/FB	+++	++	++	(+)	++

AF tissue degeneration	None	None	Testis and SG	Guts and SG	None

TF tissue degeneration	None	FB	Testis and SG	Guts and SG	None

The number of *A. marginale *colonies was evaluated for tick gut muscle (GM), Malpighian tubule (MT) and fat body (FB) sections after acquisition feeding (AF) and transmission feeding (TF). Scale: – (colonies not found), + (very rare; colonies found in < 10% sections), ++ (rare; colonies found in 10–39% sections), +++ (abundant; colonies found in > 40% sections). Tissue degeneration was evaluated for tick guts, salivary glands (sg), testis and fb after AF and TF. The findings were parenthesized or underlined when gene knockdown resulted in lower or higher colony counts, respectively when compared to controls.

**Figure 3:**
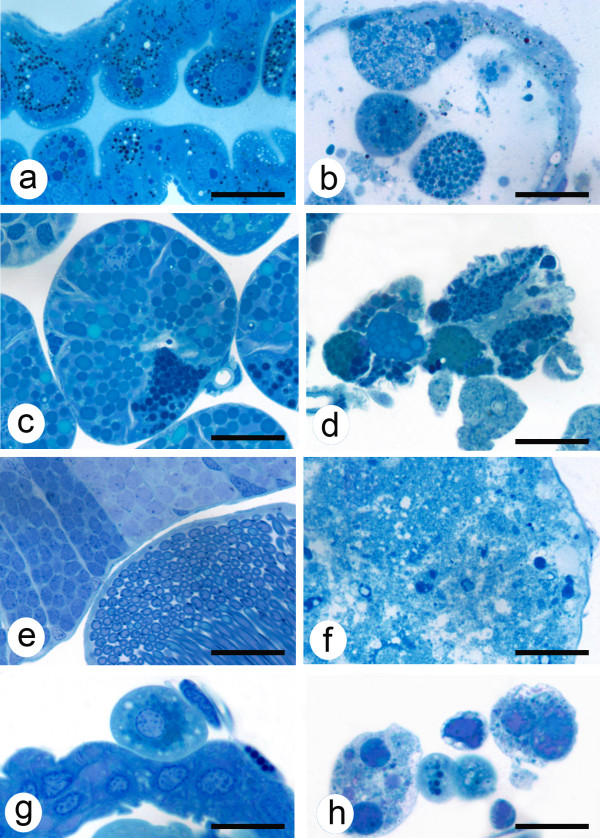
**Light micrographs of tick tissues from saline- and dsRNA-injected ticks**. Saline-injected ticks had normal gut (a), salivary gland (c), spermatogonia and prospermatids (e) and (g) fat body tissues. Tissue degeneration was observed in guts (b), salivary glands (d), spermatogonia and prospermatids (f) and/or fat body cells (h) in ticks injected with subolesin, vATPase, GST, or SelM dsRNA. Mallory's stain, bar = 10 μm.

Compared with the controls, differences in tissue degeneration were observed in the salivary glands, testis and/or guts of vATPase and subolesin dsRNA-injected ticks after AF and TF (Table 4; Fig. 3). In ticks with SelM knockdown, fat body degeneration was observed after TF (Table 4; Fig 3).

## Discussion

Previous reports documented differential gene expression in *A. marginale*-infected tick guts and salivary glands and cultured tick cells [7]. The expression of GST, SelM, vATPase and subolesin was upregulated in *D. variabilis *and/or IDE8 tick cells in response to infection with *A. marginale *[7,9]. Conversely, functional analysis of these genes by RNAi demonstrated that *A. marginale *infection levels in *D. variabilis *guts and/or salivary glands were reduced after gene knockdown [7]. However, these experiments did not provide evidence of how these genes affected the developmental cycle of *A. marginale *in ticks, which was the objective of the experiments reported herein.

The results reported in this study further confirm that GST, SelM, vATPase and subolesin are overexpressed in response to infection of ticks with *A. marginale *to increase infection/multiplication rate [7]. In general, the number of *A. marginale *colonies was lower in most tissues in gene knockdown ticks when compared to controls. Notably, colonies were not seen by light microscopy in salivary glands of the gene silenced ticks, suggesting that transmission may be diminished or prevented. The results of the light microscopy analysis further suggested that the proteins encoded by these genes have different impacts on the development of *A. marginale *in ticks. GST may be important for the development of DF in guts of AF ticks. Subolesin was also essential for the multiplication of the pathogen in gut cells both after AF and TF as gene knockdown resulted in significantly lower RF-containing colonies. The increase in the densities of RFs in SelM dsRNA-injected ticks after AF and TF and DFs in the gut of vATPase dsRNA-injected ticks after TF provided interesting results suggesting that gene silencing affected the development of the pathogen. SelM knockdown in tick guts after AF and TF resulted in higher densities of colonies containing RFs, and thus appeared to inhibit development of *A. marginale *to the dense or infective forms. However, densities of colonies containing DFs were not significantly different from the controls in guts after AF or TF.

In most cases, the results of *A. marginale *infection levels determined by *msp4 *PCR were similar to light microscopy findings of RF- and DF-containing colonies in guts and salivary glands. However, some incongruence was observed between both types of analysis. In all cases except for the number of RF-containing colonies in the gut of SelM knockdown ticks after AF and TF, the *msp4 *PCR results showed higher infection levels than those predicted by light microscopy analysis when compared to controls. The detection of higher infection levels by PCR may be explained either by the PCR amplification of DNA from organisms not forming colonies or resulted from the sampling observed in a single cross section of the tick halves. Also, PCR did not differentiate between tissues that may be dissected together while light microscopy analysis allowed for examination of individual tick tissues. In ticks with SelM knockdown, light microscopy analysis showed an increase in *A. marginale *RF but not DF-containing colonies, which may have also influenced the results obtained by both methods.

The mechanism by which these proteins affect *A. marginale *developmental cycle in ticks is still unknown. However, information on the function of these proteins can be incorporated into discussion of their role in *A. marginale *infection/multiplication. Selenoproteins are selenocysteine (Sec)-containing proteins that are involved in a variety of cellular processes such as oxidant metabolism [10]. In humans, SelM is expressed in many tissues and is localized in the endoplasmic reticulum [11]. In ticks, Ribeiro et al. [12] identified selenoproteins in salivary glands of *I. scapularis *after blood feeding or *B. burgdorferi *infection. However, little is known about the function of these proteins in ticks. In other arthropods such as *Drosophila*, selenoproteins have been implicated in survival, salivary gland development and fertility [13,14]. SelM was overexpressed in IDE8 tick cells infected with *A. marginale *and a selenoprotein gene was overexpressed in *A. marginale*-infected *R. microplus *ticks [7]. SelM was also overexpressed in the gill of white shrimp (*Litopenaeus vannamei*) infected with the white spot syndrome virus [15]. Taken together, these results suggest that selenoproteins may function to reduce the oxidative stress caused by pathogen infection in ticks. However, as shown herein, SelM may have other functions in ticks, perhaps related to salivary gland development, that explain why reduction in its expression prevents *A. marginale *from infection and/or multiplication in salivary glands after TF. The increase noted in the colony densities containing RFs in SelM silenced ticks both after AF and TF, suggests that expression of this gene directly impacts the *A. marginale *developmental cycle.

GST belongs to a gene family that functions in the detoxification of xenobiotic compounds and metabolites produced by cell oxidative stress [16-18]. GSTs have been found to be overexpressed in both infected [19,20] and uninfected ticks [21]. In human cells infected with *A. phagocytophilum *or *R. rickettsii*, GST genes were down-regulated [22,23]. GST was overexpressed both in IDE8 tick cells and *D. variabilis *salivary glands in response to infection with *A. marginale *[7]. However, congruent with proteomics results, real-time RT-PCR analysis of GST expression in *D. variabilis *guts and *R. microplus *ticks revealed that mRNA levels were higher in uninfected ticks [7]. These results suggest that ticks have multiple GST genes with different tissue-specific expression patterns that could play different roles during *A. marginale *infection [21]. As in other arthropods [24-27], GSTs may be involved in tick innate immunity by protecting cells from oxidative stress as a result of bacterial infection [18]. Additionally, GST may function as a stress response protein during blood feeding in ticks [19-21]. As determined by RNAi combined with PCR and light microscopy analysis of *A. marginale*, GST appears to be required for pathogen infection of *D. variabilis *guts and salivary glands and IDE8 cells, thus suggesting that the pathogen benefits from GST function, perhaps by diminishing the deleterious effect that cell oxidative stress metabolites may have on bacterial multiplication and development [7,28]. Most interesting in this study was the notable increase of *A. marginale *infection in fat body cells in the GST silenced ticks, which represents a change in the *A. marginale *developmental cycle.

vATPase is a multisubunit enzyme that mediates acidification of eukaryotic intracellular organelles which has been associated with the cytoskeleton and clathrin-coated vesicles that facilitate receptor-mediated endocytosis required for rickettsial infection [6,29,30]. Functional vATPase was shown to be required for the normal function of the Golgi complex, endoplasmic reticulum, vacuoles and endocytotic and exocytotic vesicles [29]. vATPase was also implicated in immunity [31]. Genetic knockout of vATPase subunits resulted in lethal phenotypes in yeast, *Neurospora*, *Drosophila *and mice [29]. The vATPase knockdown in *Drosophila *and human cells reduced influenza virus replication [32]. In ticks, vATPase has been implicated in salivary fluid secretion in *Amblyomma americanum *[33]. The results in *A. marginale*-infected tick cells were similar to those in *D. variabilis *ticks infected with *R. montanensis *in which vATPase mRNA levels were increased [7,34], as well as studies in which human HL-60 cells were infected with *A. phagocytophilum *[23]. Furthermore, RNAi of vATPase expression reduced *A. marginale *infection of *D. variabilis *gut cells but not pathogen multiplication in IDE8 cells [7]. These results together with those reported herein suggest that vATPase may be functionally important for *A. marginale *development in ticks by affecting pathogen infection of guts and salivary glands. Additionally, vATPase knockdown resulted in testis and salivary gland degeneration, suggesting a role for this molecule in the function of these organs.

The tick subolesin was recently discovered as a tick protective antigen in *Ixodes scapularis *[35]. Subolesin was shown by both RNAi gene knockdown and immunization trials using the recombinant protein to protect hosts against tick infestations, reduce tick survival and reproduction, and cause degeneration of gut, salivary gland, reproductive tissues and embryos [36-41]. Subolesin was shown to function in the control of gene expression in ticks through the interaction with other regulatory proteins [7,42,43]. These studies demonstrated a role of subolesin in the control of multiple cellular pathways by exerting a regulatory function on global gene expression in ticks. Subolesin was also shown to be differentially expressed in *Anaplasma*-infected ticks and cultured tick cells [7,42]. The targeting of tick subolesin by RNAi or immunization was also resulted in decreased vector capacity of ticks for *A. marginale *and *A. phagocytophilum*, respectively [8]. Consistent with these results, in the experiments reported herein subolesin knockdown resulted in gut and salivary gland degeneration and affected the development of both DFs and RFs in the gut and the movement to and infection of salivary glands. These results provide additional evidence of the role of subolesin during *A. marginale *developmental cycle in ticks. RNAi has become an important tool for the study of gene expression and function in ticks [44]. However, little is known about the process of RNAi in ticks [45]. In a recent study, we analyzed the possible off-target effects after tick subolesin RNAi and found that it is a highly specific process [42]. However, these studies have not been performed for other tick genes. Therefore, the possibility of off-target effects may exist particularly for multigene families such as those including SelM and GST. Nevertheless, it is likely that off-target effects, if present would affect the expression of other members of the gene family that are relevant for the results presented and discussed herein.

## Conclusion

The results of this RNAi and light microscopic analyses of tick tissues infected with *A. marginale *after the silencing of genes functionally important for pathogen development support previous findings [7] and suggest a role for these molecules during pathogen life cycle in ticks. The decrease in the number of DF-containing colonies suggests an effect of these genes in pathogen development from RFs to infective DFs with a possible decrease in pathogen transmission by ticks. The decrease in the number of RF-containing colonies suggests an effect of these genes on pathogen infection and replication in ticks. These results suggested that *A. marginale *may increase the expression of SelM and GST to reduce the oxidative stress caused by pathogen infection and thus increase pathogen multiplication in tick cells. The vATPase may be involved in pathogen infection of tick guts and salivary gland cells by facilitating pathogen infection by receptor-mediated endocytosis. For tick subolesin, the results presented herein provide further support for its role in different molecular pathways including those required for *A. marginale *infection and multiplication in ticks. Salivary gland infections were not seen in any of the gene-silenced ticks, raising the question of whether these ticks were able to transmit the pathogen. Finally, the results of these studies suggest that GST, SelM, vATPase and subolesin may be candidate antigens for use in the development of transmission-blocking vaccines for control bovine anaplasmosis.

## Methods

### Ticks

*Dermacentor variabilis *male ticks were obtained from the laboratory colony maintained at the Oklahoma State University, Tick Rearing Facility. Larvae and nymphs were fed on rabbits and adults were fed on sheep. Off-host ticks were maintained in a 12 hr light: 12 hr dark photoperiod at 22–25°C and 95% relative humidity. To obtain infected *D. variabilis*, male ticks were allowed to AF for one week on a splenectomized calf experimentally infected with the Virginia isolate of *A. marginale *when the parasitemia was ascending. The ticks were then removed and maintained off-host for 4 days and then allowed to TF for an additional week on an uninfected calf. Uninfected ticks were fed in a similar way on the uninfected control calf. Animals were housed with the approval and supervision of the Oklahoma State University, Institutional Animal Care and Use Committee.

### RNA interference in ticks and sample collection

The GST (Genbank accession number DQ224235), SelM (ES429105), vATPase (ES429091) and subolesin (AY652657) dsRNA synthesis was done using the Access RT-PCR system (Promega, Madison, WI, USA) and the Megascript RNAi kit (Ambion, Austin, TX, USA) as reported previously [7,8,38]. For RNAi, male *D. variabilis *ticks were injected with approximately 0.4 μl of dsRNA (5 × 10^10^–5 × 10^11 ^molecules per μl) in the lower right quadrant of the ventral surface of the exoskeleton. The injections were done on 50 ticks per group using a Hamilton syringe with a 1 inch, 33 gauge needle. Control ticks were injected with injection buffer (10 mM Tris-HCl, pH 7, 1 mM EDTA) alone. The ticks were held in a humidity chamber for 1 day after which they were allowed to AF and acquire infection for 7 days on a splenectomized calf that was experimentally infected with the Virginia isolate of *A. marginale *(rickettsemia during tick feeding ranged from 4.8% to 35.9% infected erythrocytes). Unattached ticks were removed two days after infestation. All ticks were removed after AF and held in a humidity chamber for four days to allow ticks to digest the bloodmeal, thus allowing for detection of only those pathogens that had infected gut cells. For studies on AF fed ticks, 10 ticks per group were cut in half, fixed and processed for light microscopy analysis. From an additional 5 ticks from each group midguts were dissected and the DNA and RNA were extracted and used to determine the *A. marginale *infection levels by *msp4 *quantitative PCR and to confirm gene expression silencing by RT-PCR. The remaining ticks were allowed to TF for 7 days on an uninfected calf to promote development of *A. marginale *in tick salivary glands. After TF, 10 ticks per group were cut in half, fixed and processed for light microscopy analysis. In addition, salivary glands and guts were dissected from 5 ticks from each group for extraction of DNA and RNA and used for determination of the *A. marginale *infection levels by *msp4 *quantitative PCR and to confirm gene expression silencing by RT-PCR.

### Confirmation of gene silencing and determination of *A. marginale *infection levels

Guts collected after AF and guts and salivary glands collected after TF were placed in RNAlater (Ambion) for extraction of DNA and RNA as previously reported [7]. *A. marginale *infection levels in ticks were determined by *msp4 *quantitative PCR as described by de la Fuente et al. [46]. Tick gut and salivary glands infections in dsRNA and saline injected ticks were compared by Student's t-test (P = 0.05). Gene expression knockdown was confirmed by determination of mRNA expression levels by real-time RT-PCR as described by de la Fuente et al. [7]. The mRNA levels were normalized against tick β-actin using the comparative Ct method and significance of gene silencing was determined by comparison of mRNA levels in dsRNA and saline injected ticks by Student's t-test (*P ≤ 0.05).

### Light microscopy and data analysis

For the microscopic studies, ticks were cut in half with a razor blade, separating the right and left sides, and the tick halves were fixed in 2% glutaraldehyde in 0.1 M sodium cacodylate buffer (pH 7.2). The tick halves were then dehydrated in a graded series of ethanol, washed and embedded in epoxy resin after Kocan et al. [47]. Thick sections (1.0 μm) were cut with an ultramicrotome, stained with Mallory's stain [48] and examined using a light microscope. A calibrated grid (each square, 0.07 mm^2^) was used to estimate the area of the gut examined in each section. The number of RF- and DF-containing colonies in the gut of each section was recorded. For the salivary glands, total number of salivary acini was tabulated in each tick section and the number of RF- and DF-containing colonies was recorded. The densities of RF- and DF-containing colonies in guts and salivary glands were determined as the number of colonies per gut mm^2 ^or salivary gland acini in each section and compared between dsRNA-injected and control ticks by Student's t-test with unequal variance (P < 0.05). *A. marginale *colonies seen in other tick tissues (gut muscle, Malpighian tubule and fat body cells) were also counted and tabulated for evaluation of the qualitative role of these tissue infections in the *A. marginale *tick developmental cycle in silenced and control ticks.

## Authors' contributions

KMK and JF conceived, designed the experiments and wrote the paper. KMK, ZZ, EB, VN, CA, and RM performed the experiments. JF and KMK analyzed the data. All authors read and approved the final manuscript.
